# Transient Neonatal Diabetes Mellitus with an Unknown Cause in a 1-Month-Old Infant: A Case Report

**DOI:** 10.3390/healthcare12131257

**Published:** 2024-06-25

**Authors:** Mateusz Tarasiewicz, Anna Pietrzykowska, Julia Włodarczyk, Sebastian Seget, Karolina Gadzalska, Paulina Jakiel, Sebastian Skoczylas, Przemysława Jarosz-Chobot, Maciej Borowiec

**Affiliations:** 1 Department of Children’s Diabetology and Pediatrics, Medical University of Silesia, 40-055 Katowice, Poland; 2 Department of Clinical Genetics, Medical University of Lodz, 90-419 Lodz, Poland

**Keywords:** transient neonatal diabetes mellitus, monogenic diabetes, insulin therapy, continuous glucose monitoring, next-generation sequencing

## Abstract

Transient neonatal diabetes mellitus (TNDM) is a genetically heterogeneous form of neonatal diabetes characterized by hyperglycemia that remits during infancy with a tendency to recur in later life. This case report presents the history of a male infant with transient neonatal diabetes mellitus. The patient was treated with a continuous subcutaneous insulin infusion (CSII) and a continuous glucose monitoring (CGM) system until the age of 2 months, when the normoglycemia connected with a withdrawal of treatment was noted. The genetic test results excluded the majority of known mutations related to TNDM. This case report focuses on various genetic mutations and the clinical features connected with them that cause TNDM and highlights the difficulties in the diagnostic and therapeutic processes of this disease. CSII and CGM systems seem to be a safe and effective treatment option in TNDM and may be used in the therapy.

## 1. Introduction

Neonatal diabetes mellitus (NDM) is an uncommon type of diabetes, typically recognized in infants under the age of 6 months at the time of diagnosis. It is mostly caused by single-gene mutations, but the diagnosis process can be challenging. According to international guidelines, every child diagnosed with diabetes within the first 12 months of life needs to be genetically tested [1]. In addition, it is crucial to rule out other types of diabetes and to ensure that there are no additional phenotypic features indicative of specific syndromes, in which elevated glucose levels can be one of the components. Additionally, pancreatic malformations should be ruled out.

In this article, we present an interesting and original case of an infant transferred from the Neonatal Intensive Care Unit to the Department of Children’s Diabetology because of hyperglycemia, with a strong suspicion of NDM. The history of the patient is an example of the various genetic causes of TNDM and highlights the challenges that occur in the process of diagnosing the etiology of the disease. The presented case also shows the necessity of making multiple adjustments in the way insulin is delivered based on the variable insulin demand in the neonatal period. This issue makes the report educational and unique.

## 2. Case Report

The male infant, at the age of 29 days and with a weight of 3270 g, was admitted to the Department of Children’s Diabetology and Pediatrics in Upper Silesian Child Health Centre in Katowice due to hyperglycemia noted in the neonatal period. The boy was born via cesarean section at 38 weeks of gestation, Apgar 10/10, with intrauterine growth retardation (IUGR), and a birth weight of 2080 g. A family history revealed gestational diabetes in the boy’s mother that has been treated with insulin, and type 2 diabetes in the patient’s paternal grandfather and aunt; however, the kind of treatment received and the age of the onset of the disease remains unknown. In the physical examination, some characteristic clinical features were found, including enlarged dimension of the braincase in comparison to the facial skeleton, macroglossia, umbilical hernia, and hydrocele in both testicles. Because of the recognition of gestational diabetes in the boy’s mother, the random plasma glucose concentration was measured in a venous blood sample within the first week after birth at the Neonatal Intensive Care Unit (NICU). Then, the hyperglycemia was detected for the first time, and the fluctuations in glycemic levels were observed, both fasting and postprandial, with the highest registered levels at 400 mg/dL (22.2 mmol/L). Arterial blood gases analysis showed the right parameters of acid-base homeostasis. Blood ketones levels were within normal limits (0.1–0.2 mmol/L). Urine analysis revealed no glycosuria or ketonuria. An infection was also ruled out as a reason for elevated blood glucose.

During the hospitalization at NICU, a swab from the patient’s cheek was taken to isolate and analyze the DNA. The genetic tests were performed by an accredited laboratory that is part of the Department of Clinical and Laboratory Genetics at the Teaching and Clinical Centre of the Medical University of Lodz. The NGS method was used to analyze the following 12 genes: *ABCC8*, *EIF2AK3*, *FOXP3*, *GCK*, *GLIS3*, *HNF1A*, *HNF1B*, *HNF4A*, *INS*, *KCNJ11*, *PDX1*, *PTF1A*. No pathogenic or potentially pathogenic variant was found to be the reason for the patient’s diabetes. The same NGS method was used to analyze the sequence of mitochondrial genome to identify potential variants responsible for monogenic diabetes, and no mutations were found. The investigation was extended to whole-exome sequencing (WES) to analyze the genes’ locus 6q24 to identify the pathogenic variants connected with the clinical phenotype of the child. In the mentioned 6q24 region, no pathogenic or potentially pathogenic variants were found. There was also no variant of uncertain significance (VUS), which could be associated with the clinical features. The occurrence of the deletion or duplication of variants in the 6q region was also excluded. The copy number variation (CNV) was also analyzed in the previously described region, and no aberrations were detected.

The test results for autoantibodies typical for type 1 diabetes were negative in the following five classes: islet cell antibodies (ICA), insulin autoantibodies (IAA), anti-tyrosine phosphatase antibodies (IA2), anti-glutamic acid decarboxylase (GAD) antibodies, and anti-Zinc transporter 8 antibodies (ZnT8). The patient’s C-peptide level was low, but it was non-diagnostic in reference to the patient’s age. The HbA1c was not marked because of the obvious high input of fetal hemoglobin (HbF) in the total hemoglobin concentration in the infant’s blood [2]. An abdominal ultrasound revealed a volumetrically small pancreas, which could be the result of the growth period. In addition, the same ultrasound showed a partial hypoplasia of the inferior vena cava and venous drainage from the lower limbs to the azygos vein system. After a few months, the diagnostic methods were used more widely at the Children’s Cardiology Department. In addition to the persistent foramen ovale found by echocardiography, other cardiovascular defects that could be components of specific NDM phenotypes were excluded.

**Figure 1:**
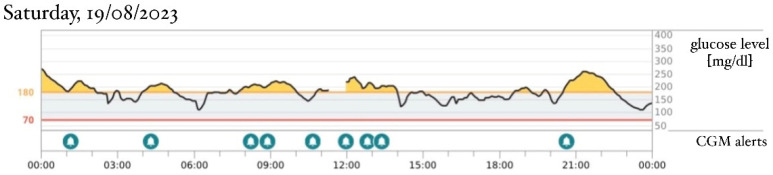
Patient’s age: 39 days, weight: 3700 g, treatment: continuous subcutaneous insulin infusion (CSII), basal infusion without boluses, lispro insulin, v = 0.06 IU/24 h.

**Figure 2:**
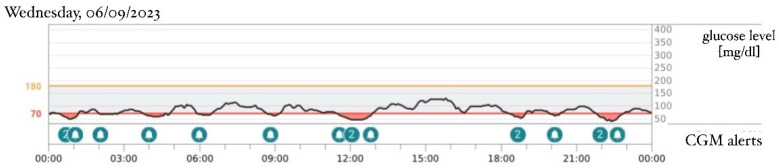
Patient’s age: 57 days, weight: 4100 g, treatment: one injection per day of ultra-long-acting insulin analog Degludec 0.5 IU per day at 8 a.m. (the day of the last injected dose).

**Figure 3:**
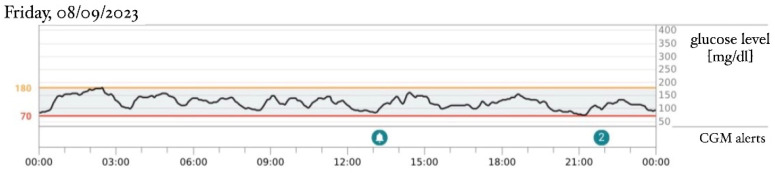
Patient’s age: 59 days, weight: 4150 g, without treatment, 48 h after the last dose of Degludec 0.5 IU.

An intravenous insulin infusion was started 2 days after hyperglycemia was detected, initiating at a dose of 0.03 U/kg/h and escalating to 0.17 U/kg/h in the next few days. There was no need to reduce the frequency of feedings. The next day after admission to the Department of Children’s Diabetology and Pediatrics, at the age of 30 days, a continuous glucose monitoring (CGM) Dexcom G6 system was implemented, and the intravenous insulin delivery was replaced by a continuous subcutaneous insulin infusion (CSII). Insulin lispro was used in the pump. The sensor-augmented pump with predictive low-glucose suspension (SAP-PLGS) and the advanced hybrid closed-loop (AHCL) systems could not be used in the treatment because of the difficulties in attaching to a compatible CGM system. Because of poor subcutaneous tissue on the arms and the abdomen, a CGM sensor was attached to one thigh and a cannula for CSII to the other thigh. The patient’s parents were educated on CSII and CGM system therapies. Basal insulin was set to the smallest dose possible to configure in the pump—0.025 IU/h (0.6 IU/24 h). The basal infusion was enough to stabilize the glycemia without the use of boluses. Due to low insulin demand according to the patient’s age and weight, an insulin dilution with 0.9% NaCl solution was applied in proportion 1:10, resulting in a dosage of 0.0025 IU/h (0.06 IU/24 h). Saline was used because of a lack of manufacturer diluent. Despite maintained glycemia levels variation (Figure 1), the practitioners considered not switching to oral sulfonylureas (SU) therapy before the results of the genetic tests were obtained, and the gradual improvement of glycemic control ultimately supported this decision. The boy gained weight properly with a diet based on mixed ways of feeding—breastfeeding and the infant formula NAN Optipro 1. Due to the weight gain and the stable insulin dosage, a reduction in the relative demand on insulin was observed. After 2 days of stable glycemia levels, the insulin therapy was modified and CSII was switched to daily subcutaneous injections of ultra-long insulin Degludec, first at a dosage of 1 and then increasing to 0.5 IU/day, using a pen injector. The boy was connected to the CGM system and discharged. He continued his insulin therapy as an outpatient, and then the CGM data were analyzed during the follow-up visit. The results showed that even the smallest dose of ultra-long insulin (0.5 IU/day) caused hypoglycemia episodes during the day, while no hyperglycemia incidents were marked in the data (Figure 2). At the age of 2 months, the insulin therapy was definitively discontinued. During a follow-up visit, the CGM data from just 2 days after the last dose of insulin Degludec were obtained and showed normoglycemia throughout the day with 100% of the time spent within a range of 70–180 mg/dL (TIR) (Figure 3). The necessity for long-term follow-up was handed over to the parents, as there is a known predisposition to diabetes in later life [3].

Figure 1, Figure 2 and Figure 3: Data from patient’s continuous glucose monitoring (CGM) Dexcom G6.

## 3. Discussion

Neonatal diabetes mellitus is a rare condition that occurs in newborns who have elevated blood glucose levels and is often detected incidentally. It is estimated that NDM accounts for 2.5–6.5% of diabetes cases in children. The occurrence of NDM is estimated in circa 1 in 20,000–350,000 births. There are two subtypes of NDM: permanent (PNDM) and transient (TNDM) neonatal diabetes. The cause of insulin deficiency in NDM comes from dysfunction of the beta-cells, destruction of their structure, or abnormal pancreatic development [1].

TNDM, which is commonly observed in neonates, generally appears during the first 6 weeks of life and remits in 3–4 months. Around 70% of the TNDM cases are associated with a mutation in chromosome 6q24, and 25% of them are caused by defects in the *KCJN11* and *ABCC8* genes, which have an influence on the activity of potassium channels at the beta pancreatic cell membranes [2]. However, the genetic basis of one-third of the cases of TNDM remains unknown [1].

Nowadays, the diagnostic methods in genetics are incredibly accurate. Genetic testing gives us the opportunity to make a proper diagnosis earlier in life, which allows us to deploy the most appropriate treatment and delay the potential complications of the disease. As an example, Karin van der Tuin et al. described an interesting case showing how modern genetics can influence the effectiveness of treatment in a patient who was misdiagnosed in earlier life [4]. According to the ISPAD guidelines, the recommended method for genetic testing in searching for NDM etiology is next-generation sequencing (NGS), which was the method used in the presented case [1]. A recently published study revealed a more efficient diagnostic process using NGS compared to the Sanger method, resulting in more appropriate treatment in specific types of NDM [5]. In the patient presented in this case report, there were some phenotypic features to which we should pay attention. Notably, the boy was presented with intrauterine growth retardation (IUGR), macroglossia, and umbilical hernia, which are the components of some specific gene mutation variants. TNDM connected with these features is typical of *PLAGL1/HYMAI* gene mutations with variable inheritance [6]. The ZFP57 gene mutation causes another type of TNDM that is also associated with congenital heart disease, such as persistent foramen ovale [7]. Both types of TNDMs are caused by mutations located on chromosome 6 at the 6q24 and 6p loci [8]. Temple I. et al. indicate in their research that in some cases of the disease there was a family history of type 2 diabetes, similar to our patient [3]. The hyperglycemia seen in the type of diabetes caused by these mutations typically does not lead to ketoacidosis and usually has its onset during the first week of life, as in the case of the described infant [9]. In their study, Docherty LE. et al. compare the clinical phenotype of 163 patients diagnosed with 6q24 TNDM. Most of the patients were born small for gestational age, and the average age of remission was around 2 months. The most frequent features were macroglossia and umbilical hernia, while congenital heart defects, e.g., persistent foramen ovale, were rare [9]. The aberrations mentioned above were excluded in the process of making the diagnosis.

An *HNF1B* mutation could also be one of the potential factors that caused the TNDM in our patient because the pancreatic hypoplasia was coexistent with diabetes, but it was excluded by the NGS test that was performed. Moreover, we could have disqualified this diagnosis before the genetic testing due to the lack of renal cysts in our patient’s ultrasound, which is one of the recognition criteria in this syndrome [10,11]. The isolated TNDM, caused by the *INS* variant, was also excluded [12].

The possibility to monitor the effects of insulin therapy is undeniably crucial for the safety of the treatment in neonates and infants, since their insulin demand is low and variable. The CGM system appears to be the most effective method to keep glycemic values under permanent control, and it has been confirmed as a dedicated way of glucose monitoring in the report of two cases of TNDM described by Chisnoiu T. et al. [13]. The use of the Dexcom G6 system, which was applied in one of the reported patients, additionally allows caregivers to react before some glycemic fluctuations happen, thanks to predictive alerts informed about glucose trends. The system also ensures permanent monitoring that displays on parents’ and caregivers’ mobile devices, which additionally increases both the safety and the comfort of the therapy.

In recent years, there have been documented cases using a continuous subcutaneous insulin infusion (CSII) in NDM treatment [14,15,16]. Our case report confirms that the CSII combined with the CGM system works successfully in children with TNDM who have unpredictable changes in their insulin demand from day to day. As we know from other research by Iwata N. et al., the CGM system is especially useful in cases of children in which a diagnosis based on blood samples is not clear. In those cases, the hyperglycemia episodes were detected only thanks to continuous monitoring [17].

In the last few years, automated insulin delivery (AID) systems have revolutionized insulin therapy with CSII. In the literature, there were some published cases presenting AID systems usage in patients with NDM. LeeMY. et al. present a case of a 2-month-old patient with NDM caused by a KCNJ11 mutation, where an AID system was implemented and thanks to that, a fluent and safe transition to oral SU was possible [18].

In such a young patient, there is a need to pay attention to poor subcutaneous tissue, which could be a problem when setting up CSII and CGM system sets. Zanfardino A. et al. report a video case which presents a pump set replacement in a very low birth weight newborn to show the challenges, but also to prove that the pump and the sensor set can be adapted even in extremely difficult cases [19].

Rabbone I. et al. published a report emphasizing the use of the CGM system for a safe transitioning from insulin therapy to oral medications like sulfonylurea in case of NDM [20]. Despite not using glibenclamide in the presented child’s therapy, it seems to be significant to mention that there is extensive proof of the successful switch from insulin therapy to oral SU medications in cases of NDM [21]. Following international guidelines, the administration of oral sulfonylurea medications is the recommended therapy in recognized NDM, particularly in cases caused by channelopathies. The use of oral SU remains a highly effective treatment in 90% of NDM cases; among them, glibenclamide has the most promising research [22]. The facts are clear-cut: prompt treatment of NDM benefits child growth and weight gain, and it is crucial for the long-term development of a child.

In the case of our patient, it was necessary to dilute the insulin in the pump to improve technical aspects of insulin delivery. In the literature, there are reported cases of usage of diluted insulin in CSII in patients with NDM. Tubiana-Rufi N. indicates that there is a frequent need to dilute the insulin in CSII in the case of patients with NDM due to a very low insulin demand [23]. In their publication, Habeb AM. et al. revealed that 80% of the 126 cases of NDM collected in their survey needed dilution of insulin to reach the adequate doses of insulin [24]. Olinder AL. et al. report the cases of two infants with NDM successfully treated with insulin pumps, in which the insulin lispro was diluted [25]. Piccini B. et al. have reported successfully treating a patient with TNDM by using diluted insulin lispro with an insulin pen injector [26].

## 4. Conclusions

Due to its uncommon prevalence, transient neonatal diabetes mellitus (TNDM) remains one of the least-researched types of diabetes. The presented case is an example of the various genetic causes of TNDM and highlights the challenges that occur in the process of diagnosing the etiology of hyperglycemia found in the postnatal period. For cases of transient diabetes, insulin therapy using CSII and CGM systems seems to remain a safe and successful treatment until the results of genetic testing are obtained, hyperglycemia is resolved, or until a decision is made to implement oral sulfonylureas. 

## Data Availability

The original contributions presented in the study are included in the article, further inquiries can be directed to the corresponding author.
